# Supplementary material for: Glycolysis on F-18 FDG PET/CT Is Superior to Amino Acid Metabolism on C-11 Methionine PET/CT in Identifying Advanced Renal Cell Carcinoma at Staging
Source: Cancers (Basel). 2021 May 14;13(10):2381. doi: 10.3390/cancers13102381 (PMC8155930; doi:10.3390/cancers13102381)
Supplement: Supplementary file 1 [file cancers-13-02381-s001.zip › cancers-1217002-supplementary.pdf]

cancers

MDPI

Article

Supplementary Material: Glycolysis on F-18 FDG PET/CT is Superior to Amino Acid Metabolism on C-11 Methionine PET/CT in Identifying Advanced Renal Cell Carcinoma at Staging

Suk Hyun Lee [1,2,†], Jee Soo Park [3,†], Hyunjeong Kim [4], Dongwoo Kim [1], Seung Hwan Lee [3], Won Sik Ham [3], Woong Kyu Han [3], Young Deuk Choi [3,*] and Mijin Yun [1,*]

Table S1. Characteristics of individual patients.

No.	Sex	Age	Tumor size	Histology	SUVmax FDG	MTV FDG	SUVmax MET	MTV MET	Fuhrman grade	AJCC stage	pT	pN	pM
1	M	86	7.2	Clear cell	6.43	28.96	7.64	4.08	3	3	3a	0	0
2	F	63	5.5	Clear cell	3.80	2.28	4.64	0.15	3	1	1b	0	0
3	M	62	5.5	Clear cell	9.94	13.00	5.46	4.40	2	4	1b	0	1
4	M	63	10.6	Papillary	6.01	295.46	4.90	148.60	3	3	3a	0	0
5	F	69	8.0	Clear cell	8.83	185.21	4.78	0.63	4	3	3a	1	0
6	M	59	8.5	Clear cell	17.73	134.92	6.18	1.54	3	3	3b	0	0
7	F	76	8.2	Clear cell	4.60	2.25	6.10	9.76	2	2	2a	0	0
8	F	79	9.0	Clear cell	7.88	67.55	10.75	16.28	3	4	3a	0	1
9	F	60	7.5	Clear cell	6.85	99.16	6.66	9.74	4	3	3a	0	0
10	F	78	7.3	Chromo-phobe	2.99	0.81	6.94	70.49	2	2	2a	0	0
11	F	76	7.0	Clear cell	3.32	0.34	4.99	21.37	2	2	2a	0	0
12	F	71	5.0	Clear cell	18.84	76.97	10.67	40.15	3	4	4	0	1
13	F	61	6.8	Chromo-phobe	2.98	1.44	4.26	0.18	2	1	1b	0	0
14	M	56	9.0	Clear cell	8.95	112.82	3.93	40.19	3	4	3a	0	1
15	M	59	11.0	Clear cell	9.21	74.40	5.25	28.87	3	3	3c	0	0
16	M	67	4.0	Clear cell	7.35	21.21	7.95	5.21	4	1	1a	0	0
17	M	35	8.5	Clear cell	3.54	0.15	1.86	0.00	2	2	2a	0	0
18	M	48	5.4	Papillary	4.06	8.35	2.85	0.00	2	1	1b	0	0

19	M	64	9.0	Clear cell	11.59	225.28	5.31	26.00	3	3	3a	0	0
20	F	52	7.0	Clear cell	8.47	31.44	4.83	3.95	3	2	2a	0	0
21	F	34	9.0	Chromo-phobe	2.23	5.52	2.81	3.57	3	2	2a	0	0
22	M	40	8.3	Clear cell	4.50	0.02	4.95	0.00	3	2	2a	0	0
23	M	56	7.7	Clear cell	2.16	0.00	1.49	1.38	2	2	2a	0	0
24	M	60	6.7	Clear cell	10.74	24.42	6.46	5.26	3	3	3a	0	0
25	M	74	8.8	Clear cell	4.55	79.91	7.61	0.79	3	3	3a	0	0
26	F	48	9.5	Clear cell	4.51	15.41	6.53	4.18	3	2	2a	0	0
27	F	66	13.0	Clear cell	7.64	515.38	9.93	424.01	3	4	4	1	1
28	F	58	13.0	Clear cell	5.96	125.38	8.99	10.48	2	4	2b	0	1
29	F	56	11.0	Clear cell	25.27	336.03	11.81	44.84	4	4	3a	1	1
30	F	68	10.1	Clear cell	3.28	0.00	1.30	0.00	2	4	2b	0	1
31	M	62	3.5	Chromo-phobe	3.18	46.91	6.82	65.99	3	3	3a	0	0
32	F	46	6.5	Clear cell	21.52	163.28	10.41	137.12	3	3	3b	0	0
33	M	39	7.7	Clear cell	2.88	0.20	2.30	0.00	2	2	2a	0	0
34	M	61	4.1	Clear cell	2.66	0.00	4.47	0.00	3	1	1b	0	0
35	M	55	9.0	Clear cell	7.39	222.17	9.24	25.78	3	4	3a	0	1
36	F	37	9.0	Clear cell	18.73	233.53	9.30	90.40	4	3	3c	0	0
37	F	57	7.0	Clear cell	3.07	2.89	6.58	1.24	2	3	3a	0	0
38	M	73	15.0	Clear cell	4.48	44.86	3.42	5.02	3	3	3a	0	0
39	M	48	10.5	Clear cell	7.19	202.94	4.72	0.07	4	3	3a	0	0
40	M	63	8.2	Clear cell	11.37	119.02	6.84	11.03	3	3	3a	0	0
